# Incidence and Outcomes Associated with Menopausal Status in COVID-19 Patients: A Systematic Review and Meta-analysis

**DOI:** 10.1055/s-0043-1772595

**Published:** 2023-12-23

**Authors:** Abolfazl Akbari, Ahmadreza Zarifian, Alireza Hadizadeh, Ezat Hajmolarezaei

**Affiliations:** 1 Mashhad University of Medical Sciences, Mashhad, Iran; 2 University Hospital Lewisham, King's College London, London, United Kingdom; 3 Tehran University of Medical Sciences, Tehran, Iran; 4 Mashhad University of Medical Sciences, Mashhad, Iran

**Keywords:** COVID-19, menopause, estrogen, climacteric, COVID 19, menopausa, estrogênio, climatério

## Abstract

**Objective** Menopause causes several changes in the body that may affect the response to COVID -19. We aimed to investigate the possible association between menopausal status and incidence and outcomes in COVID-19 patients.

**Methods** Combinations of keywords *COVID-19* , *menopause* , and *estrogen* were used to search the PubMed, Embase, Web-of-Science, and Scopus databases for articles reporting the incidence and outcomes of COVID-19 (discharge, length-of-admission, intensive care, or mortality) in premenopausal women, available through December 29, 2022. Data from studies comparing the incidence of COVID-19 infection with the age-matched male population were pooled and meta-analyzed using a random-effects model.

**Results** Overall, 1,564 studies were retrieved, of which 12 were finally included in the systematic review to compare disease outcomes, and 6 were meta-analyzed for the incidence of COVID-19 in premenopausal and postmenopausal women. All studies reported better COVID-19-associated outcomes in premenopausal women compared with postmenopausal women. After adjusting for confounding factors, three studies found better outcomes in postmenopausal women, and two found no association between menopausal status and COVID-19 outcomes. Our meta-analysis found a higher incidence of COVID-19 infection among premenopausal women than postmenopausal women, when compared with age-matched men (odds ratio = 1.270; 95% confidence interval: 1.086–1.486; *p* = 0.003).

**Conclusion** The incidence of COVID-19 was significantly higher in premenopausal women than in postmenopausal women when compared with age-matched men. Although premenopausal women may have more favorable COVID-19-associated outcomes, the presumed preventive effect of estrogens on the incidence and related outcomes of COVID-19 in premenopausal women cannot be proven at present. Further longitudinal studies comparing pre- and post-menopausal women are required to provide further insight into this matter.

## Resumo

**Objetivo** A menopausa causa diversas alterações no corpo que podem afetar a resposta ao COVID-19. Nosso objetivo foi investigar a possível associação entre o status da menopausa e a incidência e os resultados em pacientes com COVID-19.

**Métodos** Combinações de palavras-chave COVID-19, menopausa e estrogênio foram usadas para pesquisar os bancos de dados PubMed, Embase, Web-of-Science e Scopus para artigos relatando a incidência e os resultados do COVID-19 (alta, tempo de internação, tratamento intensivo cuidados ou mortalidade) em mulheres na pré-menopausa, disponível até 29 de dezembro de 2022. Dados de estudos comparando a incidência de infecção por COVID-19 com a população masculina da mesma idade foram agrupados e meta-analisados usando um modelo de efeitos aleatórios.

**Resultados** No geral, 1.564 estudos foram recuperados, dos quais 12 foram finalmente incluídos na revisão sistemática para comparar os resultados da doença e 6 foram meta-analisados para a incidência de COVID-19 em mulheres na pré e pós-menopausa. Todos os estudos relataram melhores resultados associados ao COVID-19 em mulheres na pré-menopausa em comparação com mulheres na pós-menopausa. Após o ajuste para fatores de confusão, três estudos encontraram melhores resultados em mulheres na pós-menopausa e dois não encontraram associação entre o status da menopausa e os resultados do COVID-19. Nossa meta-análise encontrou uma maior incidência de infecção por COVID-19 entre mulheres na pré-menopausa do que mulheres na pós-menopausa, quando comparadas com homens da mesma idade (odds ratio = 1,270; intervalo de confiança de 95%: 1,086–1,486; p = 0,003).

**Conclusão** A incidência de COVID-19 foi significativamente maior em mulheres na pré-menopausa do que em mulheres na pós-menopausa quando comparadas com homens da mesma idade. Embora as mulheres na pré-menopausa possam ter resultados mais favoráveis associados ao COVID-19, o efeito preventivo presumido dos estrogênios na incidência e nos resultados relacionados ao COVID-19 em mulheres na pré-menopausa não pode ser comprovado no momento. Mas estudos longitudinais comparando mulheres pré e pós-menopausa são necessários para fornecer mais informações sobre este assunto.

## Introduction

The emergence of severe acute respiratory syndrome coronavirus 2 (SARS-CoV-2)-induced Coronavirus Disease 2019 (COVID-19) has caused serious illness and death around the world. The World Health Organization (WHO) reported > 546 million confirmed cases and > 6 million deaths as of August 17, 2022. 1 Males reportedly have higher rates of disease severity, 2 hospitalization, readmission, 3 and mortality 4 compared with females with COVID-19. Following the recent reports on this sex difference, 5 researchers have tried to investigate the possible causes. In comparison with premenopausal women, postmenopausal women have considerably decreased plasma sex hormone concentrations (for example, estrogen and progesterone depletion). 6 It has long been known that estrogen plays a role in the immune response and regulates both the innate and adaptive immune systems. 7 Previous reviews have put forward possible effects of estrogen on the entrance and replication of viruses, innate/adaptive immune responses, and thrombosis. 8 The protective role of estrogen is reportedly linked to downregulating the expression of angiotensin-converting enzyme 2 (ACE-2), which acts as the SARS-CoV-2 receptor on target cells, by estradiol and modulation of the immune response. In vivo studies have also shown that estrogen treatment can reduce morbidity and mortality in mice infected with the Influenza A virus, 9 10 where higher levels of estrogen administration were associated with increased survival and lower pulmonary cytokine production after influenza infection. 10 11

Despite the growing body of evidence addressing predisposing factors for COVID-19 (for example, older age, gender, comorbidities), little is known about the association between menopausal status and COVID-19 outcomes and the role of sex hormones in COVID-19. In the present study, we systematically reviewed the available literature on the link between menopausal status and COVID-19 incidence and outcomes, comparing pre- and postmenopausal women. We also pooled data from premenopausal or postmenopausal groups that had an age-matched control group to reduce the effects of confounding factors.

## Methods

The present study followed the Preferred Reporting Items for Systematic Review and Meta-Analysis Protocols (PRISMA) standards. 12 The study was approved by the Ethics Committee of Mashhad University of Medical Sciences, Mashhad, Iran (code: IR.MUMS.IRH.REC.1402.050).

The PubMed, SCOPUS, Web of Science, and Embase databases were searched for studies investigating the relationship between menopausal status and COVID-19 infection up to December 29, 2022. We manually searched Google Scholar and the reference lists of the included papers to find other studies that might meet our inclusion criteria. The search terms *COVID-19* , *menopause* , and *estrogen* were used in various combinations.

Initially, all studies in the English language that reported information on menopausal status and COVID-19 patients were included with no restrictions on publication date. 13 After removing the duplicates, all articles comparing the incidence and outcomes of premenopausal and postmenopausal females with COVID-19 were included in the systematic review. Studies comparing the variables with age-matched males were included in the meta-analysis. Review articles, case reports, non-human studies, letters, reports based on Web sites, and government regulatory documents were all excluded.

The study selection was performed by two reviewers (Akbari A. and Hadizadeh A.) based on the title, abstract, and full text of the papers. When there was no agreement, the decision was made by a third reviewer (Zarifian A.), who checked eligibility to make the final decision. Two reviewers (Akbari A. and Hadizadeh A.) independently assessed the quality of the included papers. The Joanna Briggs Institute (JBI) assessment tools were used to assess the included papers. 14 Any disagreement was resolved by discussion between the authors.

Study characteristics including the first author's surname, publication date, title, study design, site of study (country), sample size, menopausal criteria, and patient recruitment date were extracted from the included articles. COVID-19 outcomes (discharge, intensive care unit [ICU] admission, length of hospitalization, and mortality) as well as further analysis of the initial findings were extracted and summarized in Chart 1 . Search strategies used in different databases are listed in supplementary Chart 2 .

**Chart 1:** Characteristics and outcomes of included studies

First author	Study design	Country	Patients	Date	Menopausal criteria	Sample size	Age (years old)	Crude findings	Further analysis	Corrected confounding variables
Ding et al.	Cross-sectional	China	Patients hospitalized at 3 branches of Tongji Hospital	28 January - 8 March, 2020	Amenorrhea > 1 year	1,730	60.33 ± 14.36	Severity (premenopause: 46% versus postmenopause: 58%) and clinical outcomes including discharge (premenopause: 23% versus postmenopause: 6%), remained in hospital (premenopause: 77% versus postmenopause: 92%), and death (premenopause: 0% veersus postmenopause: 2%) were significantly different between premenopausal women and postmenopausal women.	**Age-match comparison** Severity ( *p* = 0.83) and clinical outcomes ( *p* = 0.49) including discharge, remained in hospital, and death did not differ significantly between menopausal women and age-matched men, whereas premenopausal women had significantly better clinical outcomes and fewer severely ill patients than age-matched men ( *p* < 0.01 for both).	Age and comorbidities
Wang XW. et al.	Retrospective cohort	China	COVID-19 inpatients at the Taikang Tongji Hospital	15 February - 30 April, 2020	Amenorrhea > 1 year	300	65.3 ± 14.8	Postmenopausal women had higher rates of severe disease (41.7 versus 0%), bilateral pulmonary infiltration (91.7 versus 64.7%), and mortality (2.0 versus 6.0%) than premenopausal women.	**Age-match comparison** Men had higher rates of severe disease (23.7% versus 0%; *p* = 0.003) and bilateral pulmonary infiltration (86.1% versus 64.7%; *p* = 0.04) than premenopausal women. However, there was no significant difference in mortality (2.0% versus 0%; *p* = 1.00) between the 2 groups. Men and postmenopausal women had the same percentage of severe disease (32.7% versus 41.7%; *p* = 0.21), bilateral lung infiltration (86.1% versus 91.7%; *p* = 0.24), and mortality (2.0% versus 6.0%; *p* = 0.25).	Age, body mass index , comorbidities, treatment, and laboratory results
Mishra et al.	Retrospective cohort	India	Females admitted at tertiary care dedicated COVID hospital	May – August, 2020	Amenorrhea > 1 year	147	39.05 ± 15.42	Length of hospital stay (premenopause: 8.6 ± 3.9; postmenopause: 14.1 ± 8.9; *p* < 0.01), severe disease (premenopause: 7.3%; postmenopause: 23.5%; *p* < 0.01), and mortality (premenopause: 0%; postmenopause: 9.8%; *p* < 0.01) were significantly different between groups.	**Multivariate logistic regression:** Menopausal status is not associated with length of hospital stay ( *p* = 0.057) or severity progression ( *p* = 0.262).	Age, obesity, comorbidities, oxygen/ventilator requirement, hemoglobin, and neutrophil to lymphocyte ratio
O'Brien et al.	Retrospective cohort	Canada	Canadian COVID-19 dataset	Up to 27 July 2020	More than 60 years/o	101,121	NR	Women had a lower COVID -19 incidence rate than men unless they were 80 years of age or older. Premenopausal women had a lower incidence rate than men of the same age.	–	
Seeland et al.	Retrospective cohort	17 countries	Electronic health records in a TriNetX Real-World database	Up to 16 July 2020	> 50	68,466	NR	Premenopausal women had higher incidence rates than men of the same age. Mortality rates increased steadily with age in both sexes, but the increase was steeper in men at 50 years old.	–	
Sha et al.	Retrospective cohort	China	Jinan Infectious diseases Hospital in Shandong, Shandong Provincial Chest Hospital in Shandong, and Huanggang Central Hospital in Hubei	31 January - 17 April, 2020	> 55	413	57.25 ± 3.75	Postmenopausal women had a higher mortality rate than premenopausal women (5.2% versus 3.8%).	**Age-match comparison** Premenopausal women had the same in-hospital mortality rate as men of the same age (3.8 versus 4.0%, *p* = 0.918). Postmenopausal women had a considerably lower mortality rate than men in the same age group (5.2 versus 21.0%, *p* = 0.007).	Age and comorbidities
Garg et al.	Retrospective cohort	India	A COVID-19 facility	April – July, 2020	Amenorrhea > 1 year	720	60.15 ± 6.39	Postmenopausal women and men had a higher risk of death than premenopausal women (premenopause: 8.6%; postmenopause: 19.4%)	**Age-match comparison** Risk of death in men ≤ 48 years old was 12.8% and in men > 48 it was 25.9%	Age and comorbidities
Liu et al.	Retrospective cohort	China	Renmin Hospital of Wuhan University (Wuhan, China) and Xiangyang Central Hospital (Xiangyang, China)	20 January - 1 April, 2020	> 55	459	63.25 ± 3.50	Postmenopausal women had a higher risk of death and longer length of hospitalization than premenopausal women (premenopause: 3; postmenopause: 7, premenopause: 18 (9–28); postmenopause: 13 (7–24)	**Age-match comparison** The difference in incidence between men and women was not observed in patients > 55 years old. 141 patients were < 55 years old, of whom 19 died (16 men versus 3 women; *p* < 0.005). Of the 318 cases > 55 years old, 115 died (47 women versus 68 men, *p* = 0.149).	Age and comorbidities
Wang M. et al.	Retrospective cohort	China	The Central Hospital of Wuhan, Wuhan Red Cross Hospital, the Central Hospital of Enshi Tujia and Miao Autonomous Prefecture, and Lichuan People's Hospital in Hubei Province	31 December, 2019–31 March, 2020	Amenorrhea >1 year	2,501	56.18 ± 4.32	–	**Multivariable logistic regression analysis and age-match comparison** There is no significant differences between premenopausal and postmenopausal females after propensity score matching by age (odds ratio of severe disease: 0.63 [0.32–1.24] and odds ratio of death: 1.83 [0.16–21.5]).	Age and comorbidities
Ferretti et al.	Retrospective cohort	Italy	Patients hospitalized at IRCCS San Matteo Foundation (Pavia, Italy) for COVID-19	February - December, 2020	> 50	1,764	70.10 ± 4.05	Postmenopausal women had higher incidence and mortality rates than premenopausal.	**Age-match comparison** Premenopausal women had higher incidence and mortality rates than men of the same age. However, incidence and mortality rates were higher in age-matched men than in postmenopausal women.	Age and comorbidities
Costeira et al.	Retrospective cohort	United Kingdom	Female users of the COVID Symptom Study application	7 May-15 June, 2020	Amenorrhea > 1 year	152,637	53.8	Menopausal women had higher rates of predicted COVID-19 ( *p* < 0.01), but tested COVID-19 patients and severity of disease were not significantly different with postmenopausal women ( *p* > 0.05).	–	
Toure et al.	Retrospective cohort	USA	Nonpregnant women admitted to the Hospital System in Rhode Island	March 1-June 30, 2020 and July 1, 2020 - February 28, 2021	> 55	1,863	67.57 ± 18.0	Postmenopause was associated with higher mortality (OR = 8.6 [2.7–27.6]), readmission (OR = 1.5 [1.04–2.2]), severe illness (OR = 5.7 [1.3–23.9]), and longer length of hospitalization (OR 1.6 = [1.2–2.2]).		

Abbreviations: CI, confidence interval; NR, not reported; OR, odds ratio.

**Chart 2:** Search strategy

PubMed (29 December)		
Search	Query	Results
**#1** (Menopause)	(post-menopausal [title/abstract] OR postmenopausal [title/abstract] OR Postmenopause [title/abstract] OR Post-menopause [title/abstract] OR peri-menopausal [title/abstract] OR perimenopausal [title/abstract] OR peri-menopause [title/abstract] OR perimenopause [title/abstract] OR premenopausal [title/abstract] OR pre-menopausal [title/abstract] OR pre-menopause [title/abstract] OR premenopause [title/abstract] OR Climacteric [title/abstract] OR Menopause[title/abstract] OR menopausal [title/abstract] OR Menstrual [title/abstract] OR Menses[title/abstract] OR Menstruation[title/abstract] OR Hypoestrogenic[title/abstract] OR Hypo-estrogenic[title/abstract] OR Estrogenic[title/abstract])	178,597
**#2** (COVID-19)	(“severe acute respiratory syndrome coronavirus 2” OR “Wuhan coronavirus” OR “Wuhan seafood market pneumonia virus” OR “COVID19 virus” OR “COVID-19 virus” OR “coronavirus disease 2019 virus” OR “SARS-CoV-2” OR “SARS2” OR “2019-nCoV” OR “2019 novel coronavirus” OR “COVID-19” OR “2019 novel coronavirus infection” OR “COVID19” OR “coronavirus disease 2019” OR “coronavirus disease-19” OR “2019-nCoV disease” OR “2019 novel coronavirus disease” OR “2019-nCoV infection” OR “Coronavirus Infections” OR “Coronavirus Infection” OR “Infection, Coronavirus” OR “Infections, Coronavirus” OR “novel coronavirus” OR Covid* OR “sars 2”)	332,066
**Final**	**#1 AND #2**	**399**
**SCOPUS (7 October)**		
**#1** (Menopause)	TITLE-ABS-KEY(post-menopausal OR postmenopausal OR Postmenopause OR Post-menopause OR peri-menopausal OR perimenopausal OR peri-menopause OR perimenopause OR premenopausal OR pre-menopausal OR pre-menopause OR premenopause OR Climacteric OR Menopause OR menopausal OR Menstrual OR Menses OR Menstruation OR Hypoestrogenic OR Hypo-estrogenic OR Estrogenic)	291,187
**#2** (COVID-19)	TITLE-ABS-KEY(“severe acute respiratory syndrome coronavirus 2” OR “Wuhan coronavirus” OR “Wuhan seafood market pneumonia virus” OR “COVID19 virus” OR “COVID-19 virus” OR “coronavirus disease 2019 virus” OR “SARS-CoV-2” OR “SARS2” OR “2019-nCoV” OR “2019 novel coronavirus” OR “COVID-19” OR “2019 novel coronavirus infection” OR “COVID19” OR “coronavirus disease 2019” OR “coronavirus disease-19” OR “2019-nCoV disease” OR “2019 novel coronavirus disease” OR “2019-nCoV infection” OR “Coronavirus Infections” OR “Coronavirus Infection” OR “Infection, Coronavirus” OR “Infections, Coronavirus” OR “novel coronavirus” OR Covid* OR “sars 2”)	417,588
**Final**	**#1 AND #2**	**583**
**Embase (30 July)**		
**#1** (Menopause)	(post-menopausal:ti,ab,kw OR postmenopausal:ti,ab,kw OR Postmenopause:ti,ab,kw OR Post-menopause:ti,ab,kw OR peri-menopausal:ti,ab,kw OR perimenopausal:ti,ab,kw OR peri-menopause:ti,ab,kw OR perimenopause:ti,ab,kw OR premenopausal:ti,ab,kw OR pre-menopausal:ti,ab,kw OR pre-menopause:ti,ab,kw OR premenopause:ti,ab,kw OR Climacteric:ti,ab,kw OR Menopause:ti,ab,kw OR menopausal:ti,ab,kw OR Menstrual:ti,ab,kw OR Menses:ti,ab,kw OR Menstruation:ti,ab,kw OR Hypoestrogenic:ti,ab,kw OR Hypo-estrogenic:ti,ab,kw OR Estrogenic:ti,ab,kw)	244,423
**#2** (COVID-19)	(“severe acute respiratory syndrome coronavirus 2”:ti,ab,kw OR “Wuhan coronavirus”:ti,ab,kw OR “Wuhan seafood market pneumonia virus”:ti,ab,kw OR “COVID19 virus”:ti,ab,kw OR “COVID-19 virus”:ti,ab,kw OR “coronavirus disease 2019 virus”:ti,ab,kw OR “SARS-CoV-2”:ti,ab,kw OR “SARS2”:ti,ab,kw OR “2019-nCoV”:ti,ab,kw OR “2019 novel coronavirus”:ti,ab,kw OR “COVID-19”:ti,ab,kw OR “2019 novel coronavirus infection”:ti,ab,kw OR “COVID19”:ti,ab,kw OR “coronavirus disease 2019”:ti,ab,kw OR “coronavirus disease-19”:ti,ab,kw OR “2019-nCoV disease”:ti,ab,kw OR “2019 novel coronavirus disease”:ti,ab,kw OR “2019-nCoV infection”:ti,ab,kw OR “Coronavirus Infections”:ti,ab,kw OR “Coronavirus Infection”:ti,ab,kw OR “Infection, Coronavirus”:ti,ab,kw OR “Infections, Coronavirus”:ti,ab,kw OR “novel coronavirus”:ti,ab,kw OR Covid*:ti,ab,kw OR “sars 2”:ti,ab,kw)	295,859
**Final**	**#1 AND #2**	**390**
**WOS (30 July)**		
**#1** (Menopause)	(TI = (post-menopausal OR postmenopausal OR Postmenopause OR Post-menopause OR peri-menopausal OR perimenopausal OR peri-menopause OR perimenopause OR premenopausal OR pre-menopausal OR pre-menopause OR premenopause OR Climacteric OR Menopause OR menopausal OR Menstrual OR Menses OR Menstruation OR Hypoestrogenic OR Hypo-estrogenic OR Estrogenic) OR AB = (post-menopausal OR postmenopausal OR Postmenopause OR Post-menopause OR peri-menopausal OR perimenopausal OR peri-menopause OR perimenopause OR premenopausal OR pre-menopausal OR pre-menopause OR premenopause OR Climacteric OR Menopause OR menopausal OR Menstrual OR Menses OR Menstruation OR Hypoestrogenic OR Hypo-estrogenic OR Estrogenic))	158,610
**#2** (COVID-19)	(TI = (“severe acute respiratory syndrome coronavirus 2” OR “Wuhan coronavirus” OR “Wuhan seafood market pneumonia virus” OR “COVID19 virus” OR “COVID-19 virus” OR “coronavirus disease 2019 virus” OR “SARS-CoV-2” OR “SARS2” OR “2019-nCoV” OR “2019 novel coronavirus” OR “COVID-19” OR “2019 novel coronavirus infection” OR “COVID19” OR “coronavirus disease 2019” OR “coronavirus disease-19” OR “2019-nCoV disease” OR “2019 novel coronavirus disease” OR “2019-nCoV infection” OR “Coronavirus Infections” OR “Coronavirus Infection” OR “Infection, Coronavirus” OR “Infections, Coronavirus” OR “novel coronavirus” OR Covid* OR “sars 2”) OR AB = (“severe acute respiratory syndrome coronavirus 2” OR “Wuhan coronavirus” OR “Wuhan seafood market pneumonia virus” OR “COVID19 virus” OR “COVID-19 virus” OR “coronavirus disease 2019 virus” OR “SARS-CoV-2” OR “SARS2” OR “2019-nCoV” OR “2019 novel coronavirus” OR “COVID-19” OR “2019 novel coronavirus infection” OR “COVID19” OR “coronavirus disease 2019” OR “coronavirus disease-19” OR “2019-nCoV disease” OR “2019 novel coronavirus disease” OR “2019-nCoV infection” OR “Coronavirus Infections” OR “Coronavirus Infection” OR “Infection, Coronavirus” OR “Infections, Coronavirus” OR “novel coronavirus” OR Covid* OR “sars 2”))	277,897
**Final**	**#1 AND #2**	**190**

Quantitative analyses were conducted on studies reporting the incidence of COVID-19 infection among premenopausal females, postmenopausal females, and age-matched males. The incidence of COVID -19 in premenopausal women was compared with that in men of the same age, and a similar comparison was made for postmenopausal women. The odds ratio (OR) of these comparisons was calculated and reported with the 95% confidence interval (CI) in brackets. A *p-value* < 0.05 was considered statistically significant. Interstudy heterogeneity was quantitatively calculated and presented using the I ^2^ index. Due to high heterogeneity (Cochran Q < 0.05), we used the random-effects model for our meta-analysis. Sensitivity analysis was performed using fixed-effects model analyses. Potential publication bias was investigated using funnel plots, as well as the Begg and Egger test. Statistical analyses were performed using Comprehensive Meta-Analysis Software (CMA v.3, Biostat Inc., Englewood, NJ, USA).

## Results

**Fig. 1:**
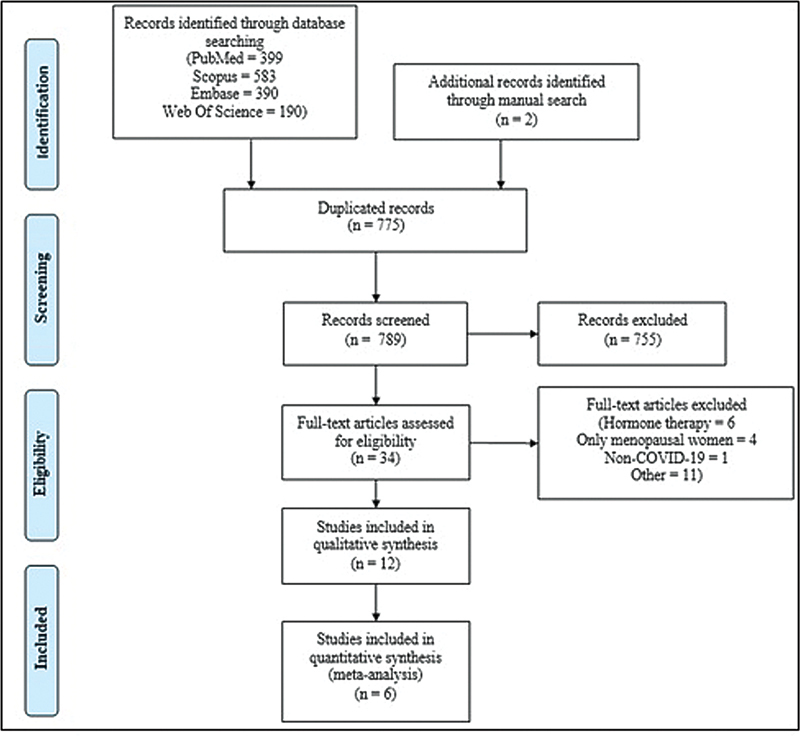
PRISMA flow chart depicts the flow of information through the different phases of the study.

A total of 1,564 studies were found by searching the databases, of which 775 were duplicates. Of the 789 remaining papers, 34 were reviewed in full text. Finally, 12 studies were included in the present systematic review 15 16 17 18 19 20 21 22 23 24 25 26 ( Fig. 1 ) and 6 were included in the meta-analysis. 15 16 17 18 19 20 21 22 23 24 25 The total number of patients in the 12 included studies was 331,821, ranging from 147 to 152,637. Seven studies were conducted in Asia (five in China 15 16 20 22 23 and two in India 17 21 ), one each in Canada, 18 United States, 25 Italy, 24 and United Kingdom, 26 and one was a multicenter study. 19 All but one cross-sectional study 15 had a retrospective cohort design.

### Incidence of COVID-19

**Fig. 2:**
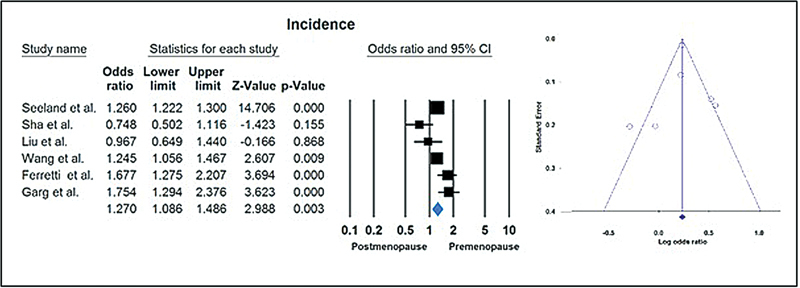
Meta-analysis of studies comparing premenopausal and postmenopausal females with age-matched males.

The meta-analysis of six studies comparing premenopausal and postmenopausal females with age-matched males showed a higher incidence of COVID-19 in premenopausal females than in postmenopausal females (OR = 1.270; 95%CI [1.086–1.486]; *p* = 0.003) ( Fig. 2 ). A sensitivity analysis for this comparison was done on two studies that used > 1 year of amenorrhea as menopausal criteria (21, 23), which showed a significantly higher incidence of COVID-19 in the premenopausal group compared with postmenopausal women (fixed effect model: OR = 1.345; 95%CI: 1.164–1.555; *p* < 0.0001). In these 6 studies, the total number of COVID-19 cases was 19,861 in the premenopausal group and 18,610 in the postmenopausal group.

### Outcomes of COVID-19

The study characteristics of 12 included studies are summarized in Table 1 . All studies reported better COVID-19-associated outcomes in premenopausal women than in postmenopausal women. However, after adjusting for confounding factors, premenopausal women had more favorable COVID-19 outcomes in only three studies (15, 16, 22), while two others found postmenopausal women to have better COVID-19 outcomes (20, 24), and three found no significant difference in this regard (17, 21, 23), while three studies did not use multivariate analysis ( Chart 1 ). The confounding factors adjusted for in each study are described in Chart 1 , with all studies adjusted for age and comorbidities. All included studies were of adequate quality ( Chart 3 ).

**Table 1:** 

Study	1. Were the two groups similar and recruited from the same population?	2. Were the exposures measured similarly to assign people to both exposed and unexposed groups?	3. Was the exposure measured in a valid and reliable way?	4.Were confounding factors identified?	5. Were strategies to deal with confounding factors stated?	6. Were the groups/participants free of the outcome at the start of the study (or at the moment of exposure)?	7. Were the outcomes measured in a valid and reliable way?	8. Was the follow-up time reported and sufficient to be long enough for outcomes to occur?	9. Was follow-up complete, and if not, were the reasons to loss to follow-up described and explored?	10. Was appropriate statistical analysis used?
Wang et al.	Y	Y	Y	N	N	Y	Y	Y	Y	Y
Mishra et al.	N	Y	Y	N	N	Y	Y	Y	Y	Y
O'Brien et al.	Y	Y	N	Y	Y	Y	Y	Y	N	Y
Seeland et al.	Y	Y	N	Y	Y	Y	Y	Y	Y	Y
Sha et al.	Y	Y	N	Y	Y	Y	Y	Y	Y	Y
Garg et al.	Y	Y	Y	N	N	Y	Y	Y	Y	Y
Liu et al.	Y	Y	N	Y	Y	Y	Y	Y	Y	Y
Wang et al.	Y	Y	Y	Y	Y	Y	Y	Y	N	Y
Ferretti et al.	Y	Y	N	Y	Y	Y	Y	Y	Y	Y
Costeira et al.	U	Y	Y	N	N	Y	Y	Y	N	Y
Toure et al. *	U	U	U	N	N	U	Y	Y	U	U

* only the abstract was available.

**Chart 3:** (B) Quality assessment table for cross-sectional studies based on JBI Critical Appraisal

Study	Were the criteria for inclusion in the sample clearly defined?	Were the study subjects and the setting described in detail?	Was the exposure measured in a valid and reliable way?	Were objective, standard criteria used for measurement of the condition?	Were confounding factors identified?	Were strategies to deal with confounding factors stated?	Were the outcomes measured in a valid and reliable way?	Was appropriate statistical analysis used?
Ding et al.	Y	Y	Y	Y	Y	Y	Y	Y

### Publication Bias

The Egger and Begg tests revealed no significant publication bias for the reported incidence rates in the included studies. Fig. 2 shows the funnel plot for the COVID-19 incidence, which also indicates no significant publication bias.

## Discussion

The meta-analysis of studies comparing premenopausal and postmenopausal females with age-matched males showed a higher incidence of COVID-19 in premenopausal women than in postmenopausal women, and the sensitivity analysis of studies that used > 1 year of amenorrhea as menopausal criteria confirmed it. We have shown that premenopausal women have better COVID-19-associated outcomes than postmenopausal women. Our findings revealed that the available literature could not still yield conclusive evidence on whether menopausal status (that is, serum estrogen levels) has a significant association with outcomes of COVID-19. Therefore, we are not able to determine if the sex-based disparities in COVID-19 incidence and outcomes is associated with estrogen levels, or if other potential effects may be influential as well. Consistent with the main finding of our study, Mishra et al. reported that most premenopausal women were more likely to have mild symptoms than postmenopausal women. 17

The differences in COVID-19-associated outcomes between premenopausal women and postmenopausal women can be explained by several factors such as age, estrogen depletion, sedentary lifestyle, and comorbidities, which are more common in postmenopausal women. 27 Some studies reported that patients affected by COVID-19 were predominantly men, 28 while some others reported both sexes as being equally affected, or women to be predominant. 29 In addition, disease severity and mortality rates were reported to be higher in men than in women. 29 However, sex differences in morbidity and mortality were less evident in patients > 70 years old when women are in postmenopausal status. 30 31 One possible justification for these findings arises from the fact that estradiol downregulates the expression of ACE-2 mRNA in bronchial epithelial cells, the host-cell receptor which has been proven to be used by SARS-CoV-2 virions for viral uptake. 32 However, our results did not confirm these findings.

The literature suggests that cytokine storm leads to adverse clinical manifestations or even acute deterioration and mortality in critically ill patients with COVD-19. 33 Impaired acquired immune responses and uncontrolled innate inflammatory responses may be associated with the mechanism of cytokine storm in COVID -19. Early control of cytokine storm by anti-inflammatory treatments may improve the survival rate of patients with COVID-19. 34 It is well-known that pretreatment of human macrophages with estrogen can reduce tumor necrosis factor alpha (TNF-α) expression by inhibiting nuclear factor-kappa B (NFk-B) and JAK2 signaling pathways. 35 Estrogen also attenuates monocyte and macrophage recruitment by downregulating the expression of chemokine ligand 2 during inflammation and dampening toll-like receptor 4-mediated NFk-B activation. 36 Along with its immunomodulatory properties, estrogen alters the expression of T helper 1 (Th-1) and Th-2 type cytokines, inhibits overactive inflammatory processes, and restores homeostatic conditions, thereby averting cytokine storm syndrome. 37 38 In a recent review, estrogens were shown to have remarkable anti-inflammatory and immunomodulatory effects on COVID-19 infections. 39 Another study showed that SARS-CoV-2 induces stress in the endoplasmic reticulum that exacerbates the infection, and estrogen may play a role in reducing the endoplasmic reticulum stress through stimulating estrogen-mediated signaling pathways. 40 An in-vivo study by Channappanavar et al. showed a protective effect of estrogen against COVID-19 death. They demonstrated that female mice given an estrogen receptor antagonist had a higher mortality rate due to SARS-COV-2 infection. Additionally, they noted that ovariectomized and gonadectomized female mice had a poor prognosis and considerable lung involvement with proinflammatory cytokines and chemokines. 41 Pirhadi et al. have also reported several antiviral effects for estrogen therapy through immunomodulatory and nonimmune mechanisms. 42 Improving the hydration of the oral cavity by stimulating hyaluronic acid production and enhancing the lower airway function can also be other probable mechanisms by which estrogen can lead to increased production of mucus-containing antiviral compounds. 43 44 In addition, estrogen therapy has been shown to decrease viral titers. 31 It may also decrease neutrophil recruitment, edema, and inducible nitric oxide synthase in the lungs. All of these have been associated with lower disease intensity. 45

The results of our meta-analysis were not inconsistent with the previous large cohort of 44,268 postmenopausal and 108,369 premenopausal women, which showed that there was no significant difference between postmenopausal and premenopausal women in terms of COVID-19 incidence. 26 Also, a cross-sectional study by Ding et al. showed a higher prevalence of COVID -19 in postmenopausal women compared with premenopausal women. 15 Costeira et al. also showed that COVID-19 patients who used oral contraceptive pills (85% of whom were premenopausal) had a lower rate of hospitalization. According to a retrospective cohort study involving 5,451 women with COVID-19, those who underwent hormone replacement therapy (HRT) had a reduced mortality risk compared with women not receiving HRT. 46 Furthermore, an important finding in the study by Seeland et al. was the strong positive effect of regular estradiol therapy on the survival of postmenopausal women with COVID-19. 19 We recommend future meta-analyses examine the role of oral contraceptive pills and hormone replacement therapy in association with COVID-19 infections. In addition, previous studies suggest that poorer COVID -19 outcome in obese patients may also be related to the level of estradiol produced by the fat mass. 47

In general, it is believed that estrogens have protective cardiovascular and metabolic effects. Studies have shown that females have a lower risk of cardiovascular events compared with males of the same age, while this risk roughly levels off after menopause. 29 48 49 Activation of G-protein-coupled receptor 30 (GPR-30) by estrogen has been shown to reduce the extent of ischemia and reperfusion injuries. 50 Reducing the low-density lipoprotein (LDL) oxidation and subsequently the oxidative stress is another reported mechanism. 51 It has also been shown that women who received HRT early after menopause had a considerably lower risk of cardiovascular events. 52 However, HRT is associated with venous thromboembolism (VTE), 53 which occurs in ∼ 15% of severe to critical COVID -19 patients. 54 Future studies should compare symptoms of COVID-19 between premenopausal and postmenopausal women to decipher the role of menopausal status and hormonal changes in COVID -19 severity.

The present study had several limitations. First, the effects of estrogen on COVID-19 outcomes may be dose-dependent, which cannot be investigated because the available studies have not assessed the serum sex hormone concentrations in COVID-19 patients. Second, postmenopausal women reportedly have higher concentrations of inflammatory cytokines compared with premenopausal women, 55 56 57 which can be a confounding factor that cannot be incorporated in our analyses. Third, the observed differences between premenopausal and postmenopausal groups are mainly due to factors such as age and comorbidities. 27 To determine the effects of sex hormones, we performed an age-matched analysis, which, however, cannot remove all confounding effects. Another limitation of the present review is the limited number of well-designed studies found with our strict inclusion criteria. Finally, some of the included studies have not used precise criteria to determine menopause in women, which can add to heterogeneity of the results.

## Conclusion

Premenopausal women have better COVID-19-associated outcomes than postmenopausal women. In addition, the incidence of COVID-19 was considerably higher in premenopausal women than in postmenopausal women when compared with age-matched men. However, the presumed preventive effects of estrogen on the incidence and outcomes of COVID-19 in premenopausal women cannot be proven at present, as other well-known risk factors that are more common in older women must also be considered. Further longitudinal studies comparing pre- and postmenopausal women are required to provide further insight into this matter.
